# Asthma

**Published:** 1901-12

**Authors:** 


**Asthma.** By Ernest Kingscote, M.B. Pp. xii., 183. London: Henry J. Glaisher. 1899.

The review of this work has, owing to unavoidable circumstances, been somewhat delayed. It is probably now too late for any review to affect the sale of the work, and our remarks upon it will therefore be brief. In our opinion the book, as a serious contribution to the pathology and treatment of asthma, is of very little value. The author, to our mind, brings forward no satisfactory proof in favour of his hypothesis that asthma is due to the pressure of a dilated heart upon the vagus. The preface contains the following extraordinary advice: "The busy practitioner, therefore, who may be unable to devote the time to these considerations, will do well to pass at once to Chapter xii., where the study of asthma proper begins." No practitioner can acknowledge himself to be not busy, and after taking the author's advice to skip the first eleven chapters, which, by the way, contain the preliminary investigations which are to establish the truth of the thesis propounded in the remaining five, we fear he is likely to skip these also.

